# Preparation of polydopamine-coated graphene oxide/Fe_3_O_4_ imprinted nanoparticles for selective removal of fluoroquinolone antibiotics in water

**DOI:** 10.1038/s41598-017-06303-y

**Published:** 2017-07-18

**Authors:** Feng Tan, Min Liu, Suyu Ren

**Affiliations:** Key Laboratory of Industrial Ecology and Environmental Engineering (MOE), School of Environmental Science and Technology, Dalian University of Technology, Dalian, 116024 China

## Abstract

Antibiotics in water have recently caused increasing concerns for public health and ecological environments. In this work, we demonstrated polydopamine-coated graphene oxide/Fe_3_O_4_ (PDA@GO/Fe_3_O_4_) imprinted nanoparticles coupled with magnetic separation for fast and selective removal of fluoroquinolone antibiotics in water. The nanoparticles were prepared by the self-polymerization of dopamine using sarafloxacin as a template. The imprinted PDA film of 10~20 nm uniformly covered the surface of GO/Fe_3_O_4_ providing selective binding sites. The nanoparticles showed rapid binding and a large capacity (70.9 mg/g). The adsorption data fitted well the Langmuir and pseudo-second order kinetic equations. The nanoparticles could be easily separated by a magnet following the adsorption and then regenerated by simple washing for repetitive adsorptions. The nanoparticles were successfully used for the removal of fluoroquinolone antibiotics in seawater, with removal efficiencies of more than 95%. The proposed strategy has potentials for efficient removal of antibiotics in environmental water.

## Introduction

Antibiotics have been extensively used to treat bacterial infections in humans and animals. In recent years, however, antibiotics in environments have caused increasing concerns for public health and ecological environments since they have been widely found in water, soil, and sediment^1, 2^. Although antibiotics in water are detected at low levels (usually μg/L–ng/L)^3^, they are continuously released into water environments, thereby imposing long-term exposure on aquatic organisms and resulting in the development of antibiotic resistance^4–6^. More than ten fluoroquinolone (FQ) antibiotics have been used for the treatment of gram-negative and gram-positive bacterial infections in humans and animals since the first FQ antibiotic norfloxacin was reported in 1979. Generally, FQs are not completely metabolized in body prior to they are released into water environments. Recently, many studies have showed that several FQ antibiotics were detected in surface water, ground water and sea water^7–9^. Thus, efficient removal of FQ antibiotics in water is very important for preventing potential threats to aquatic organisms and human health.

Various techniques have been applied to eliminate antibiotics in water, such as biodegradation^10^, photodegradation^11, 12^, membrane separation^13^, and adsorption^14–16^. Among these techniques, adsorption has many advantages, such as less energy consumption, ease of operation, and low cost of maintenance. Adsorbents play a critical role in the adsorption removal of pollutants. Common adsorbents used include activated carbon^17^, carbon nanotube^18^, graphene^19^, metal oxide^20^, sewage sludge^21^, and smectite clay^22^. However, these adsorbents possess poor selectivity and the co-adsorption of other common inorganic and/or organic compounds in water leads to low removal efficiency for target pollutants. Therefore, developing selective adsorbents is highly attractive for efficient removal of targets in water.

Molecularly imprinted polymers (MIPs) are synthetic receptors with tailor-made imprinted sites, which can selectively bind template and its analogs. In recent years, MIPs have been used as adsorbents for the removal of toxic pollutants. However, bulk MIPs prepared by traditional bulk polymerization could not provide sufficient removal efficiency due to its low adsorption capacity and poor binding kinetics. To address this problem, molecularly imprinted nanoparticles with abundant and accessible binding sites have been prepared by direct precipitation polymerization or surface molecular imprinting technology for *in-situ* polymerization on the surface of various nanomaterials^23–25^. In additional, water compatibility must be considered for the application of MIPs in aqueous environments. Despite recent progresses in the development of hydrophilic MIPs, the preparation of MIPs nanoparticles with specific recognition to targets in aqueous solutions remains challenges.

Polydopamine (PDA) has recently shown super-adhesion on various material surfaces, good environmental stability, biocompatibility, and hydrophilicity^26^. PDA is usually prepared via the self-polymerization of dopamine in weak alkaline condition. Three-dimensional imprinted sites can be achieved when a template is introduced during the self-polymerization, producing specific recognition ability for the template^7–9^. The thickness or size of PDA materials can be easily adjusted by changing the polymerization time, which provides an advantage to obtain ultrathin imprinted PDA film on the surface of nanomaterials to achieve excellent binding kinetics. For example, several PDA-based imprinted nanomaterials, such as PDA@Fe_3_O_4_ nanoparticles^27–31^, PDA@graphene^32^, PDA@multi-walled carbon nanotubes^33^, and PDA nanowires^34, 35^ have been prepared for fast separation of proteins in biological samples. Recently, magnetic PDA/graphene imprinted composite and membrane have been reported for efficient enrichment/separation of glycopeptides and methylene blue in aqueous solutions^9, 36–38^.

In the present study, we showed selective removal of antibiotics in water using PDA-coated graphene oxide/Fe_3_O_4_ (PDA@GO/Fe_3_O_4_) imprinted nanoparticles, which were prepared by the self-polymerization of dopamine in the presence of GO/Fe_3_O_4_ and sarafloxacin as a template in Tris-HCl buffer. The prepared nanoparticles were characterized, and its adsorption performance for FQ antibiotics was studied. Finally, the nanoparticles were successfully used for the removal of FQ antibiotics in seawater.

## Experimental

### Materials

Sarafloxacin (SAR), ofloxacin (OFL), gatifloxacin (GAT), enrofloxacin (ENR), ciprofloxacin (CIP), and tetracycline (TC) were purchased from Meilun Biological Technology Corp. (Dalian, China). Dopamine (DA), tris(hydroxymethyl) aminomethane hydrochloride (Tris-HCl), and phthalic acid (PA) were obtained from Aladdin (Shanghai, China). Graphene oxide (GO) power was obtained from Nanjing XFNANO Materials Tech Co., Ltd (Nanjing, China). Deionized water (DI) used was prepared by a Milli-Q® ultrapure water system (Bedford, MA USA).

### Apparatus

Transmission electron microscopy (TEM) characterizations were carried out with a FEI Tecnai G220. Infrared analyses were conducted with a Shimadzu IR-Prestige-21FTIR spectrometer. A Mettler-Toledo TGA/SDTA851 analyzer was used for thermogravimetric analysis (TGA) under N_2_ protection ranging from 20 to 800 °C at 10 °C/min. Elemental analysis was performed by an Elementar Vario EL III element analyzer. Magnetic properties of the nanoparticles were tested using a JDM-13 vibrating sample magnetometer at 300 K. The Brunauer–Emmett–Teller (BET) surface area was measured at −196 °C using a Quantachrome Autosorb S14.

### Preparation of PDA@GO/Fe_3_O_4_ nanoparticles

**Figure 1:**
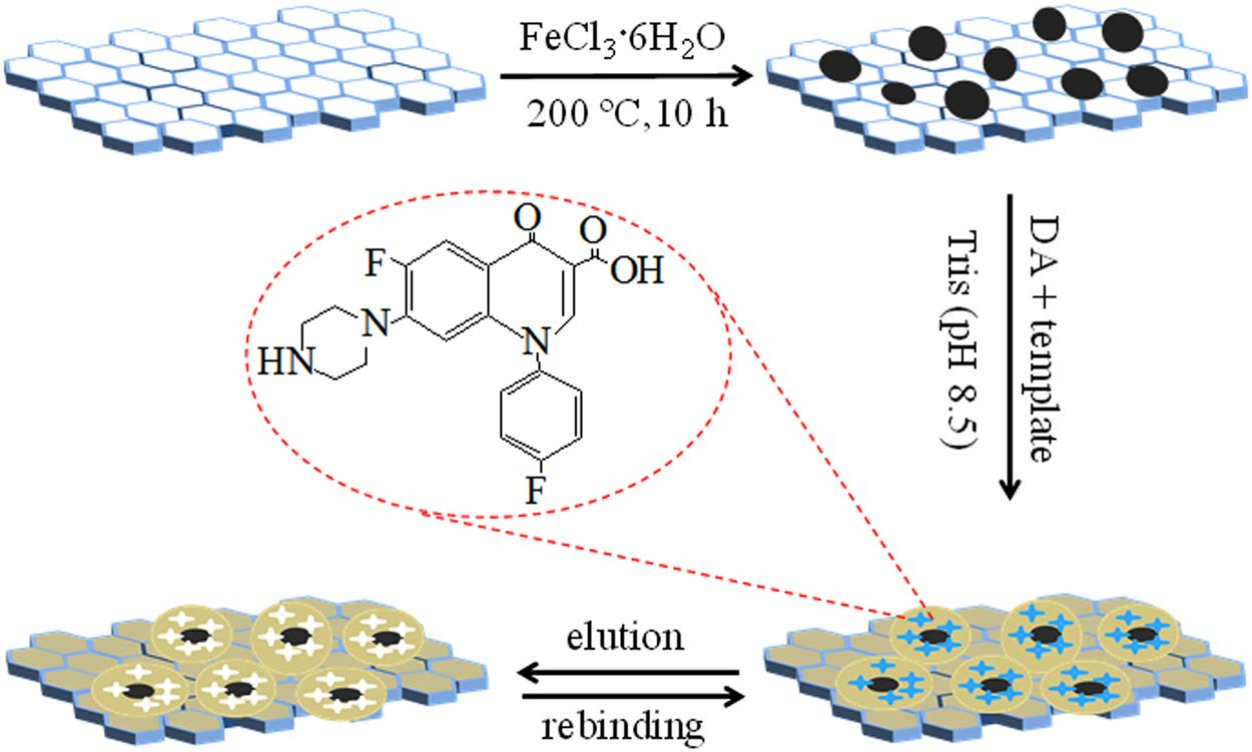
Schematic diagram for the preparation of the PDA@GO/Fe_3_O_4_ nanoparticles.

The preparation of the imprinted PDA@GO/Fe_3_O_4_ nanoparticles was shown in Fig. 1. GO/Fe_3_O_4_ nanoparticles were firstly prepared by a hydrothermal method. Briefly, 40 mg GO was dispersed in 40 g ethylene glycol under ultrasonication for 2 h. 0.2 g FeCl_3_·6H_2_O, 1.8 g sodium acetate and 0.8 g PEG-4000 were added in the GO suspension under vigorous magnetic stirring at ambient temperature for 2 h. Then the suspension was kept in a 50 mL Teflon lined autoclave reactor at 200 °C for 10 h. The resulting GO/Fe_3_O_4_ precipitate was collected, adequately washed with DI water, and dried under vacuum.

PDA@GO/Fe_3_O_4_ nanoparticles were prepared by the self-polymerization of dopamine using SAR as a template. 20 mg GO/Fe_3_O_4_ was dispersed in 50 mL ultrapure water under ultrasonication for 30 min and then mixed with 3 mg SAR, 25 mg DA and 5 mL Tris-HCl (200 mM, pH 8.5). The mixing suspension was suffered a polymerization reaction at ambient temperature for 2 h. This process resulted in a controlled and thin PDA film on the surface of the GO/Fe_3_O_4_. The resulting PDA@GO/Fe_3_O_4_ nanoparticles was removed and adequately washed with a methanol/acetic acid (v/v = 9:1), and dried under vacuum. Specific imprinted sites were exposed after the removal of the embedded template in the film. Nonimprinted PDA@GO/Fe_3_O_4_ (NPDA@GO/Fe_3_O_4_) nanoparticles were prepared by the same procedures in the absence of SAR.

### Sorption experiments

Batch sorption experiments of SAR with the nanoparticles were carried out at ambient temperature. For the adsorption capacity of the nanoparticles, 2 mg of the nanoparticles were added into 8 mL SAR standard solution ranging from 5 to 150 μM. After being shaken for 24 h, the nanoparticles were separated by a magnet, and the supernatant was analyzed by HPLC. For the binding kinetics, 2 mg of the nanoparticles and 8 mL SAR solution (60 μM) was used, and 20 μL of the supernatant was drawn respectively at 1, 3, 5, 10, 30, 60, 90, and 120 min and analyzed by HPLC. All data reported were based on triplicate experiments.

### Removal of FQs in seawater

Seawater samples were collected from Xinghai Bay, Dalian, China. The original samples didn’t contain FQs by HPLC analysis. 5 mg PDA@GO/Fe_3_O_4_ nanoparticles was added into 4 mL seawater spiked with five FQs (1 μM of each for SAR, OFL, GAT, ENR, and CIP) and incubated in a thermostatic shaker at ambient temperature for 120 min. After the adsorption, the nanoparticles were separated by a magnet, and 0.5 mL of the supernatant was used for HPLC determination.

### HPLC determination

HPLC determination was carried out by a Shimadzu Prominence LC-20A HPLC with a Shimadzu Shim-pack C18 (250 mm × 4.6 mm, 5 μm) separation column and a UV detector set at a wavelength of 294 nm. The mobile phase was water/acetonitrile (v/v = 73:27) containing 0.1% TFA with a flow rate of 0.8 mL/min. 20 μL of samples were injected for HPLC analysis. Limit of detection (LOD) and limit of quantification (LOQ) of the HPLC method for six FQ antibiotics were 0.01 µM and 0.03 µM, respectively.

## Results and Discussion

### Characterization of PDA@GO/Fe_3_O_4_ nanoparticles

**Figure 2:**
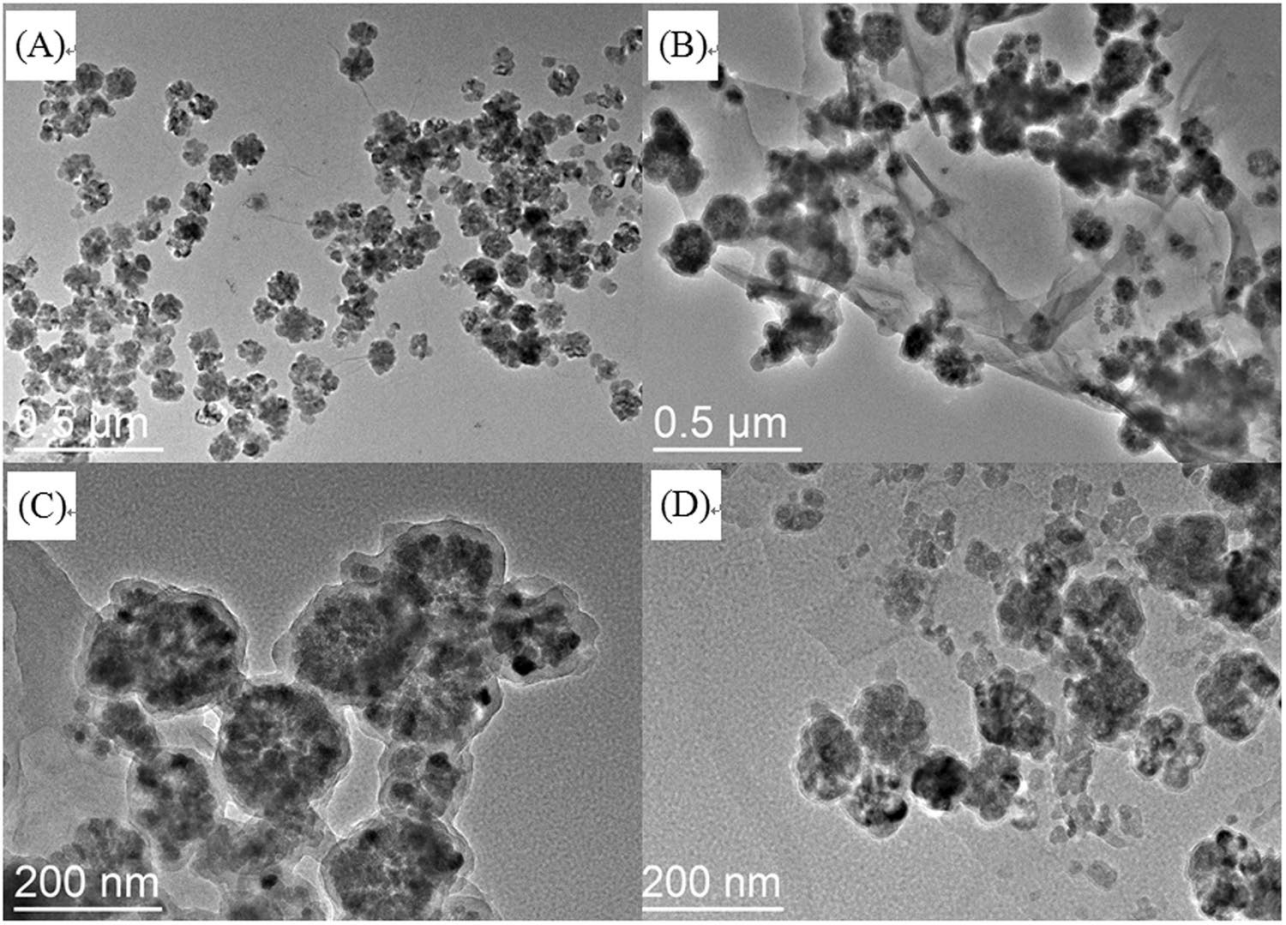
TEM images of GO/Fe_3_O_4_ (**A**), PDA@GO/Fe_3_O_4_ (**B**), PDA@GO/Fe_3_O_4_ (**C**), and NPDA@GO/Fe_3_O_4_ (**D**).

The size and shape of the prepared GO/Fe_3_O_4_ and PDA@GO/Fe_3_O_4_ nanoparticles were characterized by TEM. As shown in Fig. 2, the Fe_3_O_4_ nanoparticles had a mean diameter of 80–120 nm, which were attached to the surface of the GO (Fig. 2A). After the self-polymerization of dopamine, a uniform PDA film was clearly observed on the surface of the GO/Fe_3_O_4_ (Fig. 2B). The average thickness of the PDA film was approximately 10–20 nm based on the bar in TEM image (Fig. 2C). Figure 2D shows the TEM image of the nonimprinted NPDA@GO/Fe_3_O_4_ nanoparticles, indicating that a similar PDA film was formed on the surface of the GO/Fe_3_O_4_ in the absence of the template.

The infrared spectra of the GO/Fe_3_O_4_, PDA@GO/Fe_3_O_4_, NPDA@GO/Fe_3_O_4_, and PDA were examined (Fig. S1A). There was an absorption peak at 570 cm^−1^, which corresponded to the stretching vibration of Fe-O. The stretching vibrations of C=O and C=O of GO contributed the two peaks at 1720 cm^−1^ and 1596 cm^−1^, respectively^37, 39^. The wide peak at approximately 3300 cm^−1^ was ascribed to the stretching vibration of the –NH_2_ group^32^. Elemental analysis showed that the contents of N in the PDA@GO/Fe_3_O_4_ and NPDA@GO/Fe_3_O_4_ were 1.91% and 2.02%, respectively, while no elemental N was found in the GO/Fe_3_O_4_, as shown in Table S1. These results confirmed a PDA film was formed on the GO/Fe_3_O_4_ surface after the self-polymerization.

TGA was applied to measure the content of the imprinted PDA film on the surface of the GO/Fe_3_O_4_ through the difference of the thermal stabilities between the PDA film and GO/Fe_3_O_4_. Both the PDA@GO/Fe_3_O_4_ and NPDA@GO/Fe_3_O_4_ displayed an abrupt weight loss between 680 °C and 690 °C (Fig. S1B), indicating a rapid decomposition of the PDA film at that temperature, while the GO/Fe_3_O_4_ showed a gradual weight loss with increasing temperature due to the decomposition of the groups containing oxygen. According to the abrupt weight loss, the calculated content of the imprinted PDA film was approximately 21.2% of the whole weight of the PDA@GO/Fe_3_O_4_ nanoparticles, which agreed well with that (20.8%) of the elemental analysis.

Generally, adsorbents with large surface areas have high adsorption capacities. The S_BET_ values of the GO/Fe_3_O_4_, PDA@GO/Fe_3_O_4_, and NPDA@GO/Fe_3_O_4_ nanoparticles were 40.33, 50.34 and 46.32 m^2^/g, respectively, by the N_2_ adsorption/desorption isotherms (Fig. S1C). The NPDA@GO/Fe_3_O_4_ had a larger S_BET_ value than that of the GO/Fe_3_O_4_. The S_BET_ value of the PDA@GO/Fe_3_O_4_ further increased compared with the NPDA@GO/Fe_3_O_4_ (Table S2), which was ascribed to the contribution of the imprinted sites in the PDA film.

Fast separation of adsorbents from sample solutions after the adsorption is necessary for real applications. Here magnetic separation technique was used to collect the PDA@GO/Fe_3_O_4_ nanoparticles in water, thus magnetic property of the nanoparticles was studied. The magnetization curves of the GO/Fe_3_O_4_ and PDA@GO/Fe_3_O_4_ showed typical superparamagnetic properties (Fig. S1D). The calculated saturation magnetizations were 37.1 and 35.6 emu/g, respectively. The similar saturation magnetizations indicated that the imprinted PDA film on the GO/Fe_3_O_4_ surface had no effect on its original magnetic property. This large saturation magnetization allowed for quick and complete separation of the nanoparticles by an external magnetic field after adsorption.

### Adsorption characteristics

#### Sorption isotherms

**Figure 3:**
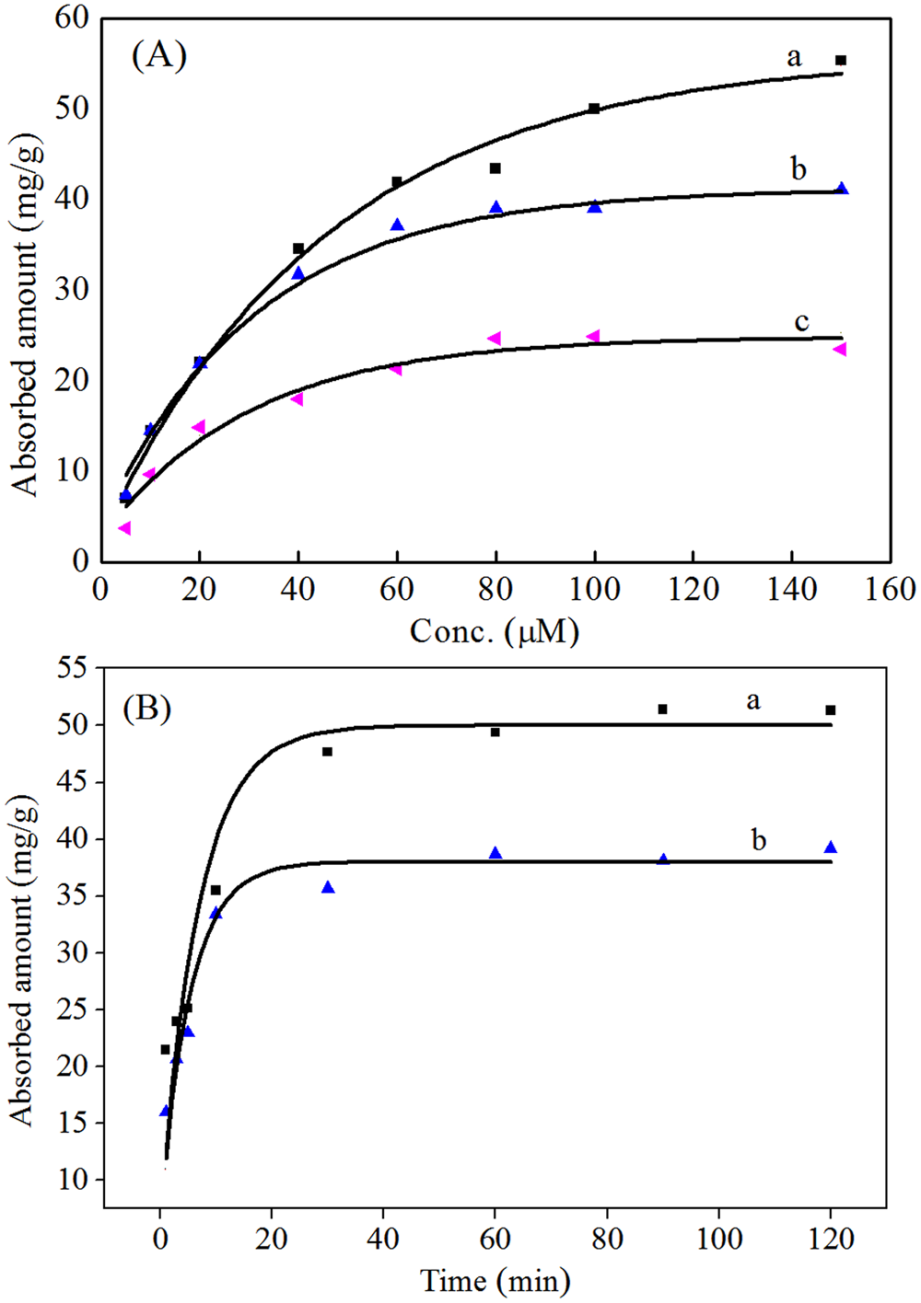
(**A**) The adsorption isotherms of SAR on the PDA@GO/Fe_3_O_4_ (a), NPDA@GO/Fe_3_O_4_ (b), and GO/Fe_3_O_4_ (c) nanoparticles. Symbols: experimental data; lines: Langmuir model predictions. (**B**) The sorption kinetics of SAR on the PDA@GO/Fe_3_O_4_ (a) and NPDA@GO/Fe_3_O_4_ (b) nanoparticles. Symbols: experimental data; lines: pseudo second order kinetic model prediction.

The sorption isotherms of SAR on the GO/Fe_3_O_4_, PDA@GO/Fe_3_O_4_ and NPDA@GO/Fe_3_O_4_ nanoparticles are shown in Fig. 3A. The adsorbed amount had an obvious increase with the increase of SAR concentration. Both the Langmuir and Freundlich adsorption models were widely used to explain the adsorption process of compounds in gas or liquid phases onto solid adsorbents. The experimental data were applied to the two model equations. The parameters calculated by the equations are given in Table 1. The Langmuir model gave larger r^2^ values ( > 0.98) than the Freundlich model. The prediction results (lines) by the former matched well with the experimental data (dots). Because the Langmuir model is based on the assumption of monolayer coverage, the nanoparticles were mainly covered by a monolayer of SAR. The maximum capacities (*q*_*m*_) calculated by the Langmuir equation were 28.0 mg/g, 47.8 mg/g, and 70.9 mg/g for the GO/Fe_3_O_4_, NPDA@GO/Fe_3_O_4_ and PDA@GO/Fe_3_O_4_ nanoparticles, respectively. The binding of the GO/Fe_3_O_4_ and NPDA@GO/Fe_3_O_4_ nanoparticles for SAR was attributed to the hydroxyl and/or amino groups through hydrogen-bonding interaction. The binding ability was further enhanced after the introduction of the imprinted sites, resulting in a larger adsorption capacity (70.9 mg/g) of the PDA@GO/Fe_3_O_4_ nanoparticles. This maximum capacity was lower than the capacity of hundreds of milligrams of common activated carbons^17^, but it was larger than that of most previous MIP materials for antibiotics, as shown in Table S3. Thus, this is an obvious improvement in the development of MIPs for the adsorption removal of toxic pollutants.

**Table 1:** The fitting parameters of the Langmuir and Freundlich equations for the adsorption of SAR on the PDA@GO/Fe_3_O_4_, NPDA@GO/Fe_3_O_4_, and GO/Fe_3_O_4_ nanoparticles.

Adsorbents	Langmuir model^a^				Freundlich model^b^	
	b (L/mg)	Q_0_ (mg/g)	r^2^	n	K_F_ [mg/g(L/mg)1/n]	r^2^
PDA@GO/Fe_3_O_4_	16.42	70.92	0.9950	1.62	5.62	0.9642
NPDA@GO/Fe_3_O_4_	8.03	47.85	0.9960	2.05	7.24	0.9274
GO/Fe_3_O_4_	8.18	28.01	0.9870	2.25	4.77	0.8649

Langmuir equation: $${C}_{e}/{q}_{e}=1/({q}_{m}\cdot b)+{C}_{e}/{q}_{m}$$ (1)

Freundlich equation: $$\mathrm{lg}\,{q}_{e}=\,\mathrm{lg}\,{K}_{F}+\mathrm{lg}\,{C}_{e}/n$$ (2)

where *q*_e_ and *q*_m_ were the amount (mg/g) of SAR on the nanoparticles at equilibrium and the maximum capacity, respectively. *C*_*e*_ was the equilibrium concentration of SAR. *b* and *K*_*F*_ were the Langmuir and Freundlich constants, respectively, and *n* was the Freundlich exponent.

#### Binding kinetics

The binding kinetic curves of the PDA@GO/Fe_3_O_4_ and NPDA@GO/Fe_3_O_4_ nanoparticles for SAR were shown in Fig. 3B. The adsorbed amounts of SAR on the nanoparticles rapidly increased with the incubation time within the initial 20 min and reached constant values after another 5 min for the NPDA@GO/Fe_3_O_4_ and 10 min for the PDA@GO/Fe_3_O_4_. The large sorption rates in the initial period indicated that there were a lot of available binding sites in the nanoparticles. The present equilibrium time was shorter than that of most reported MIPs, as shown in Table S3. The short equilibrium time was ascribed to the good dispersal abilities of the nanoparticles in water and the excellent binding characteristic of SAR deriving from the accessible binding sites in the nanoparticles. The PDA@GO/Fe_3_O_4_ had a longer equilibrium time than that of the NPDA@GO/Fe_3_O_4_, which could be ascribed to the further transport of SAR through the imprinted sites in the PDA film into the binding sites of the interior GO/Fe_3_O_4_.

The experimental data were applied to the equations of the pseudo first and pseudo second order models. The parameters calculated by the equations are shown in Table 2. The equation (4) gave better correlation coefficients (r^2^ > 0.999) than the equation (3). The prediction results (lines) by the equation (4) agreed well with the experimental data (dots) (Fig. 3B), indicating the adsorption of SAR on the nanoparticles followed up the pseudo second order model where the chemisorption was a rate-limiting step^23^. The adsorption capacities (*q*_*e*_) calculated from the fitting results were 53.2 mg/g and 39.8 mg/g for the PDA@GO/Fe_3_O_4_ and NPDA@GO/Fe_3_O_4_ nanoparticles, respectively.

**Table 2:** Kinetic parameters of the pseudo-first-order and pseudo-second-order equations for the adsorption of SAR on the PDA@GO/Fe_3_O_4_ and NPDA@GO/Fe_3_O_4_ nanoparticles.

Adsorbents	Pseudo-first-order			Pseudo-second-order			
	k_1_	q_e_(Cal.)	r^2^	k_2_	q_e_(Cal.)	v_0_	r^2^
PDA@GO/Fe_3_O_4_	0.0192	25.972	0.849	0.0049	53.191	13.89	0.9992
NPDA@GO/Fe_3_O_4_	0.0204	15.292	0.806	0.0097	39.841	15.33	0.9995

Pseudo-first-order equation: $\mathrm{ln}({q}_{e}-{q}_{t})=\,\mathrm{ln}\,{q}_{e}-{k}_{1}t$ (3)

Pseudo-second-order equation: $t/{q}_{t}=1/({k}_{2}\cdot {q}_{e}^{2})+t/{q}_{e}=1/{v}_{o}+t/{q}_{e}$ (4)

where *q*_*e*_ and *q*_*t*_ were the amount (mg/g) of SAR on the nanoparticles at equilibrium and *t* (min), respectively, *k*_*1*_ (min^−1^) and *k*_*2*_ (mg·g^−1^·min^−1^) were the pseudo first and pseudo second order rate constants, respectively, and *v*_*0*_ was the initial rate.

#### pH of solution

**Figure 4:**
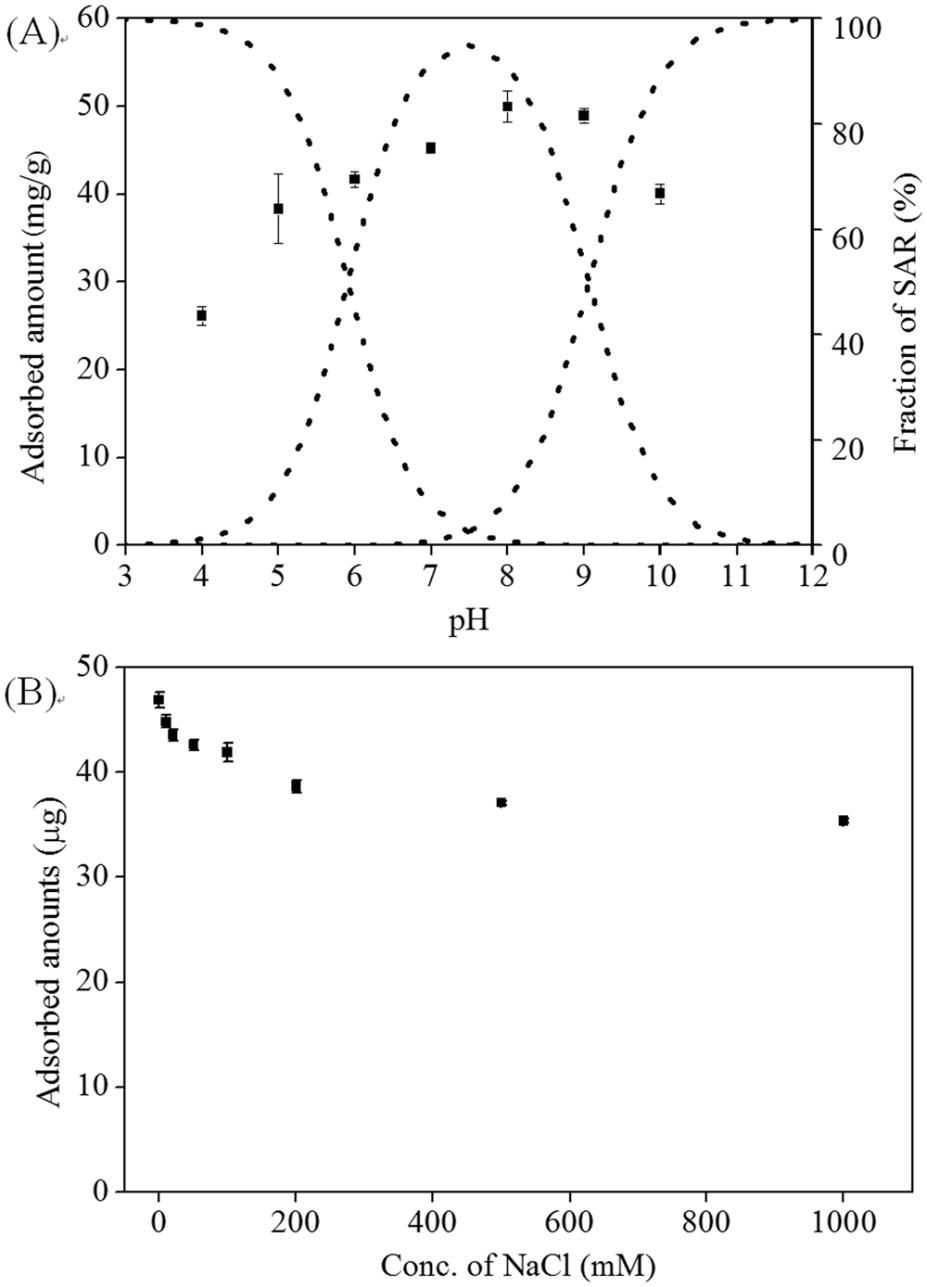
(**A**) Effect of pH on the adsorbed amount of SAR on the PDA@GO/Fe_3_O_4_ nanoparticles (squares) and fraction of SAR at different pH (dots), (**B**) effect of ionic strength on the adsorbed amount of SAR on the nanoparticles.

pH of solution usually needs to be considered in adsorption study because it not only influences the surface charges of adsorbents but also affects the ionization of compounds in aqueous solutions. It had been reported that the *pKa1* and *pKa2* values of SAR were 5.91 and 9.07^40^, which corresponded to the carboxyl group at position 3 and the amino group of the 7-piperazinyl ring, respectively, and that the *pKa* value of dopamine was 8.93^41^. Figure 4A shows the adsorbed amount of SAR on the PDA@GO/Fe_3_O_4_ nanoparticles and the fraction of various SAR species at different pH. The largest adsorbed amount was obtained at pH 8.0. At this pH value, most of SAR presented as neutral molecules, and a little fraction of SAR were anions (Fig. 4A), while the PDA film contained many positive charges. Therefore, the adsorption was mainly ascribed to the molecular recognition of the binding sites for SAR. In addition, the electrostatic interaction between SAR and the PDA film contributed to the adsorption. At pH less than 4.0, the PDA film contained more positive charges, and SAR presented as cations since the amino groups ionized. Therefore, the decreased molecular recognition and electrostatic repulsion resulted in a low binding amount. Similarly, at high pH (greater than ∼9), the PDA film was electric neutrality and SAR presented mainly as anions, and the adsorption was mainly attributed to the molecular recognition, thus achieving a relative low binding amount. Therefore, the solution pH should be maintained at 8.0~9.0 to obtain large binding amount.

#### Ionic strength of solution

The effect of salt concentration on the adsorption of SAR on the nanoparticles was investigated. Results showed that the adsorbed amount of SAR had an obvious decrease with the increase of NaCl concentration ranging from 1 to 200 mM and then maintained a constant value though the concentration continually increased, as shown in Fig. 4B. Some studies showed that adding salts enhanced the adsorption of compound on adsorbents, while others drew opposite results. The inconsistent conclusions reflected the complexity of the effect, which might involve in screening effects, electrostatic interactions, or enhanced solubility^42, 43^. Here the adsorbed amount decreased following the addition of NaCl, which could be attributed to the decrease electrostatic interaction between SAR and the PDA film due to the screening effect of the salt. When the salt concentration was sufficiently high, the electrostatic interaction reduced even disappeared completely, at which time the molecular recognition based on hydrogen-bonding interaction became dominant for the adsorption of SAR on the nanoparticles, thus the adsorbed amount remained a constant value.

#### Cross-reactivity

**Figure 5:**
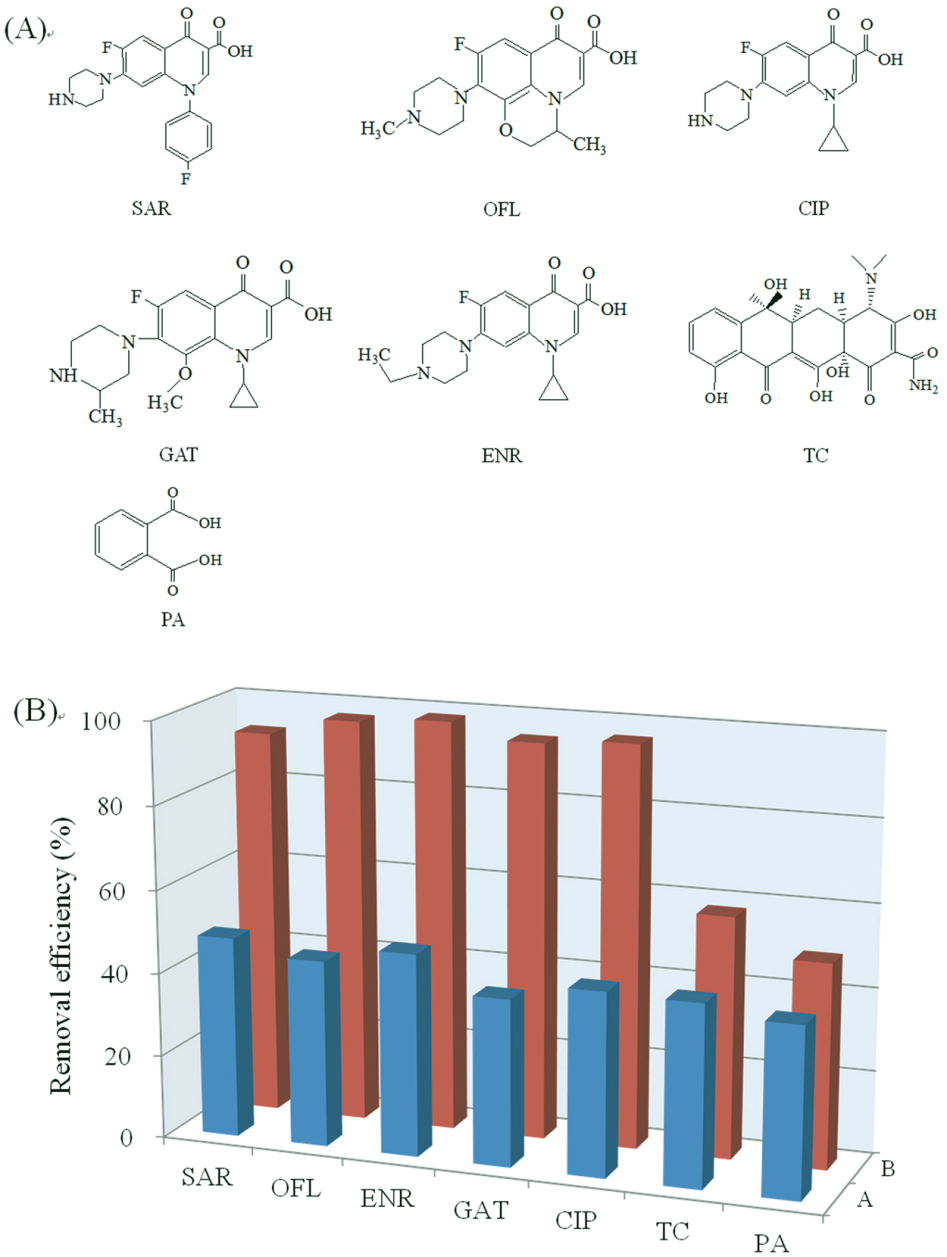
(**A**) Molecular structure of five FQs (SAR, OFL, CIP, GAT, EBR), TC, and PA. (**B**) removal efficiencies of the NPDA@GO/Fe_3_O_4_ (**A**) and PDA@GO/Fe_3_O_4_ (**B**) for five FQs (SAR, OFL, GAT, ENR, and CIP) and TC and PA.

**Figure 6:**
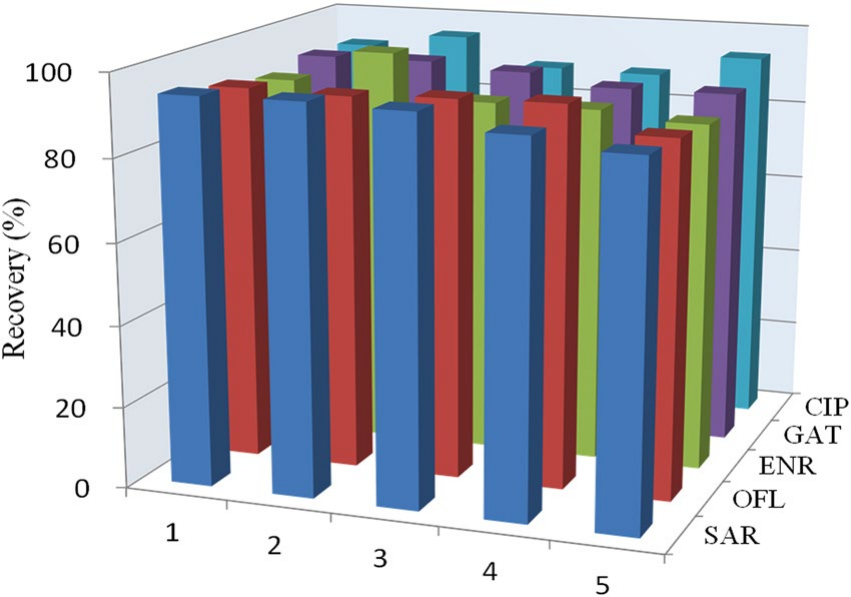
Removal efficiencies of the five FQ antibiotics in seawater by the PDA@GO/Fe_3_O_4_ nanoparticles for five adsorption-regeneration cycles.

The main aim of the present study was to develop an efficient method for fast and selective removal of FQ antibiotics in water, the adsorption of the PDA@GO/Fe_3_O_4_ nanoparticles to five FQ antibiotics (SAR, OFL, GAT, ENR, and CIP), tetracycline and phthalic acid was evaluated. The molecular structures of the compounds are shown in Fig. 5A. The five FQs have the same molecular structure except for functionality differences at 4- and 10- positions, while tetracycline and phthalic acid simulating interfering compounds have large differences from the FQs’ structure. As shown in Fig. 5B, the PDA@GO/Fe_3_O_4_ nanoparticles exhibited higher removal efficiencies (more than 95%) for the five FQs than that for tetracycline and phthalic acid. This result indicated that the nanoparticles could selectively recognize the compounds with molecular structures similar to the template SAR. This advantage allows selective removal of FQ antibiotics in the presence of other compounds. Although the present selectivity is undesirable for the detection of single FQ, it provides an advantage in the field of water treatment where different FQ antibiotics need to be simultaneously removed. The adsorption of the nonimprinted NPDA@GO/Fe_3_O_4_ nanoparticles for the compounds was evaluated. Results showed that the NPDA@GO/Fe_3_O_4_ produced lower removal efficiencies for the FQs (less than 50%) compared with the PDA@GO/Fe_3_O_4_ (Fig. 6B). And the similar removal efficiencies for the FQs and tetracycline and phthalic acid indicted that the adsorption with the imprinted NPDA@GO/Fe_3_O_4_ was dominated by nonspecific interactions.

#### Selective removal of FQs in seawater

Several antibiotics have been detected in seawater^9^. Compared with surface water and ground water, removing antibiotics in seawater is difficult due to high concentration salts and relative high pH, which deteriorates the adsorption performance of adsorbents. The present PDA@GO/Fe_3_O_4_ nanoparticles showed the largest capacity at pH 8.0, which is very close to pH (7.8~8.2) of seawater, thus we used seawater samples to evaluate the feasibility of the nanoparticles for the removal of FQ antibiotics. One µM FQs spiked seawater was treated by the nanoparticles. After the adsorption, the nanoparticles were washed with methanol/acetic acid solution for repetitive adsorptions. Figure 6 shows the removal efficiencies of the nanoparticles for the five FQ antibiotics in five consecutive adsorption-regeneration cycles, indicating without any loss of removal efficiency, and the average removal efficiencies were more than 95%. This result indicated the prepared PDA@GO/Fe_3_O_4_ nanoparticles coupled with magnetic separation were feasible for efficient removal of FQ antibiotics in seawater. And the demonstrated reusability presents an advantage over some traditional adsorbents such as activated carbon.

## Conclusion

In conclusion, magnetic PDA@GO/Fe_3_O_4_ imprinted nanoparticles have been prepared for selective removal of FQ antibiotics in water by specific recognition and magnetic separation. The nanoparticles showed a large adsorption capacity (70.9 mg/g) and excellent sorption kinetics deriving from the abundant binding sites and rapid transfer of FQ molecules in the nanoparticles. The adsorption potential was mainly ascribed to the molecular recognition and electrostatic interactions between the molecules and the PDA film. The nanoparticles could be facilely separated by a magnet following the adsorption and was regenerated by simple washing. The nanoparticles could be used repeatedly for the removal of FQ antibiotics in seawater without loss of removal efficiency. The proposed method has potentials for efficient removal of antibiotics in environmental water.

## Electronic supplementary material

Supporting Information
