# Supplementary material for: Preparation of polydopamine-coated graphene oxide/Fe3O4 imprinted nanoparticles for selective removal of fluoroquinolone antibiotics in water
Source: Sci Rep. 2017 Jul 18;7:5735. doi: 10.1038/s41598-017-06303-y (PMC5515973; doi:10.1038/s41598-017-06303-y)
Supplement: Supplementary file 1 — Supporting Information [file 41598_2017_6303_MOESM1_ESM.pdf]

Supporting Information

Preparation of polydopamine-coated graphene oxide/Fe_3O_4 imprinted nanoparticles for selective removal of fluoroquinolone antibiotics in water

Feng Tan,[a,*], Min Liu, Suyu Ren

[†]Key Laboratory of Industrial Ecology and Environmental Engineering (MOE), School of Environmental Science and Technology, Dalian University of Technology, Dalian 116024, China

Corresponding Author:

Dr. Feng Tan: Tel/Fax: 86-411-84707965, e-mail: tanf@dlut.edu.cn,

Table S1 Elemental analysis of the GO/Fe_3O_4, $PDA@GO/Fe_3O_4$, and $NPDA@GO/Fe_3O_4$

Elements	Elemental content (%)		
	$PDA@GO/Fe_3O_4$	$NPDA@GO/Fe_3O_4$	GO/Fe_3O_4
N	1.91	2.09	0.00
C	29.11	22.85	21.06
H	1.42	1.37	1.816

Table S2 The surface area and total pore volume data obtained from Brunauer–Emmett–Teller measurements

Samples	S_{BET}/m^2g^{-1}	V_P/cm^3g^{-1}	D_{BJH}/nm
$PDA@GO/Fe_3O_4$	50.34	0.121	3.804
$NPDA@GO/Fe_3O_4$	46.32	0.189	3.816
GO/Fe_3O_4	40.33	0.151	3.768

Table S3 The adsorption capacities of molecularly imprinted polymers for antibiotics in water.

Adsorbents	compounds	Adsorption capacity (mg/g)	Equilibrium time (min)	Ref
Ag@SiO_2-MIP nanoparticles	tetracycline	3.8		[1]
thermosensitive Fe_3O_4@MIP nanoparticles	norfloxacin	52.8	150	[2]
hollow MIP nanorods	chloramphenicol	37.1–62.2	120	[3]
Fe_3O_4@MIP nanorods	sulfamethazine	37.6	90	[4]
GO/MIP composite	tetracycline	21.3–40.8	100	[5]
temperature-responsive MIP particles	sulfadiazine	7.7	20	[6]
MIP particles	tetracycline	4.2	/	[7]
Fe_3O_4@MIP particles	rhodamine B	2.3	30	[8]
silica-imprinted nanoparticles	sulfamethazine	21.2	30~120	[9]
yeast@MIP particles	ciprofloxacin	14.5–21.1	120	[10]
MIP microspheres	Tetracycline	14.9–25.8	240	[11]
MIP microspheres	erythromycin	79.1	/	[12]
magnetic halloysite nanotubes@MIP	tetracycline	26.3	30	[13]
MIP-inorganic hybrid particles	tetracycline	58.2	/	[14]
MIP microspheres with brushes	sulfamethazine	10.7–22.8	120	[15]
MIP with Zn center particles	enrofloxacin	1.1	/	[16]
mesoporous carbon @MIP nanoparticles	luoroquinolone antibiotics	41.0	120	[17]
thermal-responsive	sulfamethazine	2.9–3.3	/	[18]

Fe_3O_4@MIP particles				
Fe_3O_4@MIP nanoparticles	tetracycline	12.5	240	[19]
MIP	sulfamethoxazole	18.0	15	[20]
MIP particles	b-lactam antibiotics	<40	/	[21]
PDA@GO/Fe_3O_4	fluoroquinolone	70.9	30	This work

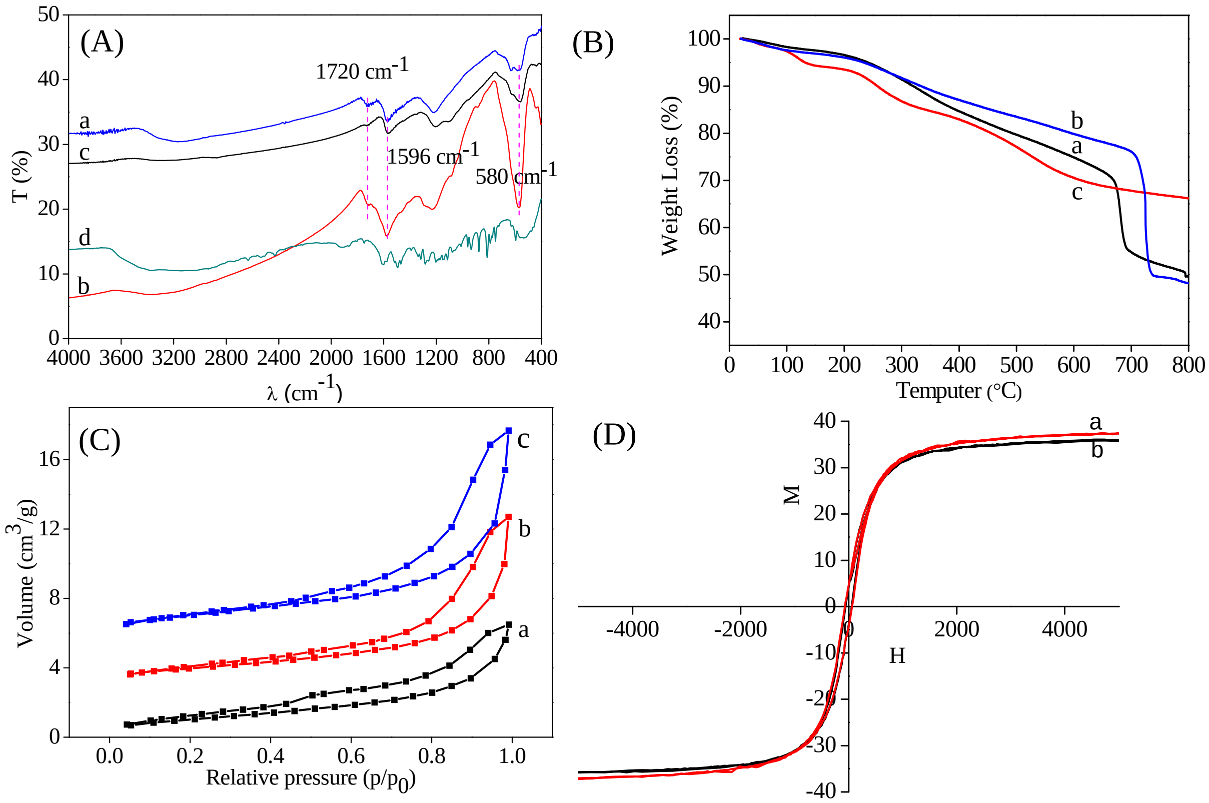

Figure S1. (A) FTIR spectra of the PDA@GO/Fe_3O_4 (a), NPDA@GO/Fe_3O_4 (b), GO/Fe_3O_4 (c), and PDA (d). (B) TGA of the PDA@GO/Fe_3O_4 (a), NPDA@GO/Fe_3O_4 (b) and GO/Fe_3O_4(c) at a heating rate of 10 ^{o}C/min from 20 to 800 ^{o}C under a N_2 protection. **(C)** Nitrogen sorption isotherms of the PDA@GO/Fe_3O_4 (a), GO/Fe_3O_4 (b), and NPDA@GO/Fe_3O_4 (c). (D) Magnetization curves of the GO/Fe_3O_4 (a) and PDA@GO/Fe_3O_4(b)

References

1. Aguilar-Garcia, D.; Ochoa-Teran, A.; Paraguay-Delgado, F.; Diaz-Garcia, M. E.; Pina-Luis, G., Water-compatible core-shell Ag@SiO_2 molecularly imprinted particles for the controlled release of tetracycline. *Journal of Materials Science* **2016,** *51* (12), 5651-5663.

2. Huang, W. H.; Kong, Y.; Yang, W. M.; Ni, X. N.; Wang, N. W.; Lu, Y.; Xu, W. Z., Preparation and characterization of novel thermosensitive magnetic molecularly imprinted polymers for selective recognition of norfloxacin. *Journal of Polymer Research* **2016,** *23* (5).

3. Xie, A. T.; Dai, J. D.; Chen, X.; Zou, T. B.; He, J. S.; Chang, Z. S.; Li, C. X.; Yan, Y. S., Hollow imprinted polymer nanorods with a tunable shell using halloysite nanotubes as a sacrificial template for selective recognition and separation of chloramphenicol. *RSC Advances* **2016,** *6* (56), 51014-51023.

4. Ma, P.; Zhou, Z. P.; Dai, J. D.; Qin, L.; Ye, X. B.; Chen, X.; He, J. S.; Xie, A. T.; Yan, Y. S.; Li, C. X., A biomimetic Setaria viridis-inspired imprinted nanoadsorbent: green synthesis and application to the highly selective and fast removal of sulfamethazine. *RSC Advances* **2016,** *6* (12), 9619-9630.

5. Liu, D.; Song, N. Z.; Feng, W.; Jia, Q., Synthesis of graphene oxide functionalized surface-imprinted polymer for the preconcentration of tetracycline antibiotics. *RSC Advances* **2016,** *6* (14), 11742-11748.

6. Ma, P. F.; Zhou, Z. P.; Yang, W. M.; Tang, B. Q.; Liu, H.; Xu, W. Z.; Huang, W. H., Preparation and application of sulfadiazine surface molecularly imprinted polymers with temperature-responsive properties. *Journal of Applied Polymer Science* **2015,** *132* (15).

7. Sanchez-Polo, M.; Velo-Gala, I.; Lopez-Penalver, J. J.; Rivera-Utrilla, J., Molecular

imprinted polymer to remove tetracycline from aqueous solutions. *Microporous and Mesoporous Materials* **2015,** *203*, 32-40.

8. Liu, X. Y.; Yu, D.; Yu, Y. C.; Ji, S. J., Preparation of a magnetic molecularly imprinted polymer for selective recognition of rhodamine B. *Applied Surface Science* **2014,** *320*, 138-145.

9. Zou, T. B.; Zhou, Z. P.; Dai, J. D.; Gao, L.; Wei, X.; Li, C. X.; Guan, W. X.; Yan, Y. S., Preparation of silica-based surface-imprinted core-shell nanoadsorbents for the selective recognition of sulfamethazine via reverse atom transfer radical precipitation polymerization. *Journal of Polymer Research* **2014,** *21* (8).

10. Wang, J.; Dai, J. D.; Meng, M. J.; Song, Z. L.; Pan, J. M.; Yan, Y. S.; Li, C. X., Surface molecularly imprinted polymers based on yeast prepared by atom transfer radical emulsion polymerization for selective recognition of ciprofloxacin from aqueous medium. *Journal of Applied Polymer Science* **2014,** *131* (11).

11. Zhao, C. Y.; Dai, J. D.; Zhou, Z. P.; Dai, X. H.; Zou, Y. L.; Yu, P.; Zou, T. B.; Li, C. X.; Yan, Y. S., One-pot method for obtaining hydrophilic tetracycline-imprinted particles via precipitation polymerization in ethanol. *Journal of Applied Polymer Science* **2014,** *131* (7).

12. Zhang, Y. X.; Qu, X.; Yu, J. P.; Xu, L. C.; Zhang, Z. Q.; Hong, H.; Liu, C. S., C-13 NMR aided design of molecularly imprinted adsorbents for selectively preparative separation of erythromycin. *Journal of Materials Chemistry B* **2014,** *2* (10), 1390-1399.

13. Dai, J. D.; Wei, X.; Cao, Z. J.; Zhou, Z. P.; Yu, P.; Pan, J. M.; Zou, T. B.; Li, C. X.; Yan, Y. S., Highly-controllable imprinted polymer nanoshell at the surface of magnetic halloysite nanotubes for selective recognition and rapid adsorption of tetracycline. *RSC Advances* **2014,**

4 (16), 7967-7978.

14. Lv, Y. K.; Zhang, J. Q.; He, Y. D.; Zhang, J.; Sun, H. W., Adsorption-controlled preparation of molecularly imprinted hybrid composites for selective extraction of tetracycline residues from honey and milk. *New Journal of Chemistry* **2014,** *38* (2), 802-808.

15. Dai, J. D.; Zou, Y. L.; Zhou, Z. P.; Dai, X. H.; Pan, J. M.; Yu, P.; Zou, T. B.; Yan, Y. S.; Li, C. X., Narrowly dispersed imprinted microspheres with hydrophilic polymer brushes for the selective removal of sulfamethazine. *Rsc Advances* **2014,** *4* (4), 1965-1973.

16. Mota, J. J. R.; Bernad, M. J. B.; Mayoral-Murillo, J. A.; Mora, J. G., Synthesis and characterization of molecularly imprinted polymers with metallic zinc center for enrofloxacin recognition. *Reactive & Functional Polymers* **2013,** *73* (8), 1078-1085.

17. Tan, F.; Sun, D. M.; Gao, J. S.; Zhao, Q.; Wang, X. C.; Teng, F.; Quan, X.; Chen, J. W., Preparation of molecularly imprinted polymer nanoparticles for selective removal of fluoroquinolone antibiotics in aqueous solution. *Journal of Hazardous Materials* **2013,** *244*, 750-757.

18. Xu, L. C.; Pan, J. M.; Dai, J. D.; Li, X. X.; Hang, H.; Cao, Z. J.; Yan, Y. S., Preparation of thermal-responsive magnetic molecularly imprinted polymers for selective removal of antibiotics from aqueous solution. *Journal of Hazardous Materials* **2012,** *233*, 48-56.

19. Dai, J. D.; Pan, J. M.; Xu, L. C.; Li, X. X.; Zhou, Z. P.; Zhang, R. X.; Yan, Y. S., Preparation of molecularly imprinted nanoparticles with superparamagnetic susceptibility through atom transfer radical emulsion polymerization for the selective recognition of tetracycline from aqueous medium. *Journal of Hazardous Materials* **2012,** *205*, 179-188.

20. Valtchev, M.; Palm, B. S.; Schiller, M.; Steinfeld, U., Development of

sulfamethoxazole-imprinted polymers for the selective extraction from waters. *Journal of Hazardous Materials* **2009,** *170* (2-3), 722-728.

21. Urraca, J. L.; Hall, A. J.; Moreno-Bondi, M. C.; Sellergren, B., A stoichiometric molecularly imprinted polymer for the class-selective recognition of antibiotics in aqueous media. *Angewandte Chemie-International Edition* **2006,** *45* (31), 5158-5161.
